# Variation of Genetic Diversity in a Rapidly Expanding Population of the Greater Long-Tailed Hamster (*Tscherskia triton*) as Revealed by Microsatellites

**DOI:** 10.1371/journal.pone.0054171

**Published:** 2013-01-17

**Authors:** Laixiang Xu, Huiliang Xue, Mingjing Song, Qinghua Zhao, Jingping Dong, Juan Liu, Yu Guo, Tongqin Xu, Xiaoping Cao, Fusheng Wang, Shuqing Wang, Shushen Hao, Hefang Yang, Zhibin Zhang

**Affiliations:** 1 College of Life Sciences, Qufu Normal University, Qufu, Shandong, People’s Republic of China; 2 State Key Laboratory of Integrated Management on Pest Insects and Rodents in Agriculture, Institute of Zoology, Chinese Academy of Sciences, Beijing, People’s Republic of China; 3 Institute of Laboratory Animal Science, Chinese Academy of Medical Sciences(CAMS) & Comparative Medicine Centre, Peking Union Medical College (PUMC), Beijing, People’s Republic of China; 4 Wugong Station for Pest Monitoring and Forecasting, Raoyang County, Hebei Province, People’s Republic of China; Auburn University, United States of America

## Abstract

Genetic diversity is essential for persistence of animal populations over both the short- and long-term. Previous studies suggest that genetic diversity may decrease with population decline due to genetic drift or inbreeding of small populations. For oscillating populations, there are some studies on the relationship between population density and genetic diversity, but these studies were based on short-term observation or in low-density phases. Evidence from rapidly expanding populations is lacking. In this study, genetic diversity of a rapidly expanding population of the Greater long-tailed hamsters during 1984–1990, in the Raoyang County of the North China Plain was studied using DNA microsatellite markers. Results show that genetic diversity was positively correlated with population density (as measured by % trap success), and the increase in population density was correlated with a decrease of genetic differentiation between the sub-population A and B. The genetic diversity tended to be higher in spring than in autumn. Variation in population density and genetic diversity are consistent between sub-population A and B. Such results suggest that dispersal is density- and season-dependent in a rapidly expanding population of the Greater long-tailed hamster. For typically solitary species, increasing population density can increase intra-specific attack, which is a driving force for dispersal. This situation is counterbalanced by decreasing population density caused by genetic drift or inbreeding as the result of small population size. Season is a major factor influencing population density and genetic diversity. Meanwhile, roads, used to be considered as geographical isolation, have less effect on genetic differentiation in a rapidly expanding population. Evidences suggest that gene flow (Nm) is positively correlated with population density, and it is significant higher in spring than that in autumn.

## Introduction

Population genetic diversity has been thought to be essential to ensure population viability [1], [2]. Genetic diversity of a population can be influenced by many factors such as dispersal, genetic drift, mutation and selection [3]–[8]. Under demographically stable conditions, the genetic diversity of populations tends to be stable and reach equilibrium between gene flow and genetic drift [9]. However, due to human disturbance such as hunting, habitat destruction or pollution, the population size of some species often declines dramatically. Reduced population size or isolation may cause genetic losses from inbreeding [10], or genetic drift [11], or even both. Mutation rates are very low, i.e. between 5.10^−3^ and 5.10^−5^ by generation, for microsatellite markers [12]. Therefore, it takes a long time to re-establish genetic diversity through mutation alone, which is not considered as one of main factors influencing genetic diversity in short-term.

Season is a major factor influencing natural selection because it determines critical environmental conditions for animals, such as food availability. In addition, results show that the population density in autumn is significantly higher than in spring. This phenomenon could be explained by several processes. First, the population has higher mortality rate in winter due to lower temperature and food shortage, which results in smaller populations the following spring. In order to mate in spring, individuals must disperse farther; Second, the population also has low breeding potential in spring due to poorer health status from poor quality food the previous winter, exacerbated by farther dispersal to mate. This situation is counterbalanced in autumn, and so the population has higher genetic diversity and lower density in spring than the ones in autumn.

Many small rodent species often experience dramatic fluctuations in population density over time or space, which may also cause variations in genetic diversity of these populations [13]. Using RAPD markers, Xie and Zhang (2006) found genetic diversity decreased with population decline in the Greater long-tailed hamster (*Tscherskia triton*) [14]. Dong *et al* (2010) found that genetic diversity of this species is positively correlated to population density using microsatellite markers [13]. Both of studies are based on data collected during the low-density phase of hamster population dynamics (i.e. monthly trap success <10%). This correlation could be explained by the following process: at high population density, individual fitness is reduced by factors such as mate competition, narrowed home range, or resource shortage, which may lead to increased dispersal. This situation is counterbalanced at low population density by genetic drift or inbreeding as the result of small population size. It is not clear if the positively correlation between the population density and the genetic diversity still works in rapidly expanding populations.

Because of the dramatically increased intensity of land use, habitat fragmentation increases the probability of local extinctions by destroying effective metapopulation structures. Habitat fragmentation especially caused by transport infrastructure has gained importance during the last couple of decades [15]. For many species, roads are barriers to dispersal owing to physical obstacles such as fences, mortality caused by the collision with vehicles or deterrence by the unknown artificial habitat [16]. Large roads have been considered as the significant barriers to gene flow, which may lead to a loss of genetic variability in fragmented populations [15], [17]. But the role of roads for dispersal of the Greater long-tailed hamster is still unclear. In this study, sub-population A and B were geographically isolated by Jingjiu railway and S282 Road, gene flow and genetic differentiation were examined to analyze the effect of roads to dispersal.

The Greater long-tailed hamster (*T. triton)* is widely distributed in the North China Plain. The general ecology of this species has been well investigated [18]. It is often considered a rodent pest to crop production in high-density years. Its population density varies greatly both seasonally and yearly. It usually breeds from early April to August with populations reaching peak in autumn (September to November) within a year. Typically for a solitary species, aggression is very high when two individuals encounter. When density is high, dispersal rate is also high due to frequent intra-specific attacks [13]. Our previous studies have indicated that genetic diversity of this species was positively associated with its population density [13], [14].

The Greater long-tailed hamster was revealed to undergo a rapidly expanding period in this area in the middle 1980’s (our unpublished data). The maximum yearly average trap success is greater than 20%, and the maximum monthly trap success is over 40%. The purposes of this study were: (1) to examine if the positive association between genetic diversity and population density still holds in the rapidly expanding population of the species during 1984 to 1990; (2) to compare the genetic diversity of the population in spring with the one in autumn; (3) to analyze genetic differentiation and gene flow between sub-population A and B. Hence, we expected to figure out the affects of population density, season and roads on genetic diversity for a rapidly expanding population of the Greater long-tailed hamster.

## Materials and Methods

### Ethics Statement

All hamster procedures were reviewed and approved by Institutional Animal Care and Use Committee of the Institute of Zoology, Chinese Academy of Sciences (Permit Number: IOZ11012). All researchers and students had been received appropriate training and certified before performing animal studies.

### Sampling Sites

**Figure 1:**
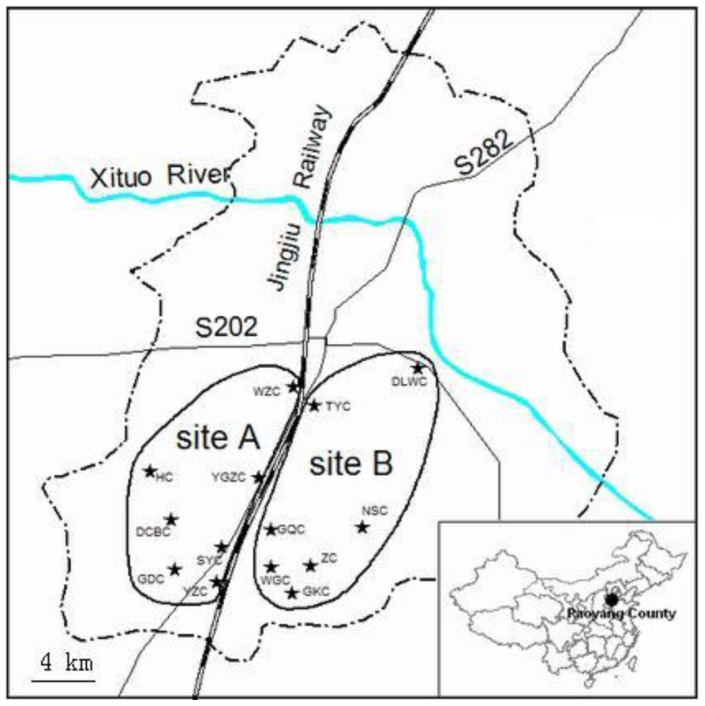
The sampling sites in the croplands of Raoyang county, China. Site A and B are isolated by Jingjiu railway and S282 Road north to south.

All samples were obtained from the Zhibin laboratory in the Institute of Zoology. Hamsters in this study were collected between 1984 and 1990 from Raoyang County (38°08′N, 115°41′E), Hebei Province, China. We defined these samples as three populations: the whole population, sub-population A and sub-population B (Figure 1). The whole population is the sum of sub-population A and B. Two sub-populations are defined based on a boundary of Jingjiu railway and S282 Road north to south. The Jingjiu railway and S282 Road bear more traffic, which were hypothesized to be the potential barriers to dispersal (Figure 1). The samples were divided into spring and autumn populations for every year between 1984 and 1990. Spring populations refer to samples collected in spring (March, April and May), and autumn populations collected in autumn (September, October and November). The study site belongs to the North China Plain. This region has a typically warm-temperate climate, with over 70% precipitation in summer (June, July and August), with very hot summers and cold winters (December, January and February). Wheat, corn, soybean, peanut and cotton are major crops of this region. The hamsters prefer to eat peanut, corn, soybean and wheat. They store many crop seeds in autumn for overcoming food shortage in winter.

### Sampling Collection

Since 1984, a monthly trapping program has been conducted for studying long-term population dynamics of this species in Raoyang County. Individuals were captured using wooden kill-traps. The wooden kill-traps were made using the principle of leverage. A piece of wood 20 cm in length and 12 cm in width is baited with peanuts and equipped with spring device. When the spring is set up and the Greater long-tailed hamsters step on the food plate to eat, the spring releases, and the hamsters are clipped, then euthanized by CO_2_ asphyxiation immediately. These operations were approved by the Institute of Zoology, Chinese Academy of Sciences. Captured hamsters were given a unique number, sexed, weighed, dissected, and measured to provide estimates of body size, age, and reproductive condition [13]. More than 20 permanent plots were selected in each sampling site, covering most crop types. Each plot contained two trapping lines spaced 25–30 m apart. Twenty-five traps were placed along each line at an interval of 5 m between each trap. Trapping was conducted for 5 days at the same time each month for 3 months followed by 3 months of non-trapping to minimize the effects of removing too many animals. The head, skeletons, liver, and stomach tissues were kept in formalin solution for later use. Population densities were defined as the percent trap success (T%). Trap success will be used as a surrogate for density throughout this manuscript. It is calculated as: T% = total number of captured hamsters/total number of traps × 100%.

Tissues were fixed in 90% ethanol, preserved in formalin solution and kept in the animal ecological Laboratory, Institute of Zoology, Chinese Academy of Sciences for more than 20 yrs. The genomic DNA was extracted from liver tissues using an improved phenol-chloroform extraction method [19].

### Genetic Analysis

One to three adult individuals were collected randomly from every sampling village in both spring and autumn 1984–1990. The sum of the samples is fifteen in spring or autumn populations 1984–1990 from site A and B. The total number of samples from site A and B in both spring and autumn 1984–1990 was 360. The liver tissues (defined as three populations: whole population, sub-population A and B as in Figure 1) were used in this study. Because the samples were very rare from 1989 to 1990, samples were pooled from these years (defined as Year 1989–1990). Genetic variation was assessed using 10 di-nucleotide repeat microsatellite loci isolated from the Greater long-tailed hamster. The loci were GYA66, GYA136, GYA183, GYA189, GYB13, GYB47, GYA185, GY103, GYB28, and GYA181, respectively(Table S1) [20].

Polymerase Chain Reactions contained 50 mM KCl, 10 mM Tris-HCl, 2.5 mM MgCl_2_, 0.2 mM each dNTP, 1 U of *Taq* DNA polymerase (Promega), 10 pM forward and reverse primers, and approximately 2 ng of template DNA in 25 µL. Amplifications began with a 5-min denaturing step at 94°C, followed by 30–35 cycles of the following thermal reaction: denaturing at 94°C for 45 s, annealing at the appropriate temperature for 45 s, and extending at 72°C for 1 min, with a final extension for 5 min at 72°C. All products were analyzed on an ABI 377 instrument (Perkin-Elmer Applied Biosystems, Foster City, California) and gel analysis was performed using GENESCAN3.1 (Perkin-Elmer Applied Biosystem).

Data were analyzed using SPSS 15.0 software (SPSS Inc.,Chicago, IL, USA). Probability of significant deviation from Hardy-Weinberg equilibrium for each locus in each population was tested using the algorithm by Levene (1949) in POPGENE VERSION 1.31 [21]. Population genetic diversity was estimated over all loci within each population by the observed average number of alleles per locus (*Na*), the effective average number of alleles per locus (*Ne*) [22], Shannon’s Information index (*I*) [23], unbiased estimates of expected heterozygosities (*He*) [24], and observed heterozygosities (*Ho*). The Pearson’s correlations between genetic diversity and population densities were analyzed by using *SPSS 15.0*. Paired t-tests were carried out to compare genetic diversities and population densities between in spring and autumn populations by using *SPSS 15.0*. Gene flow [25] and genetic differentiation between sub-population A and B were carried out by POPGENE VERSION 1.31 [21].

## Results

**Figure 2:**
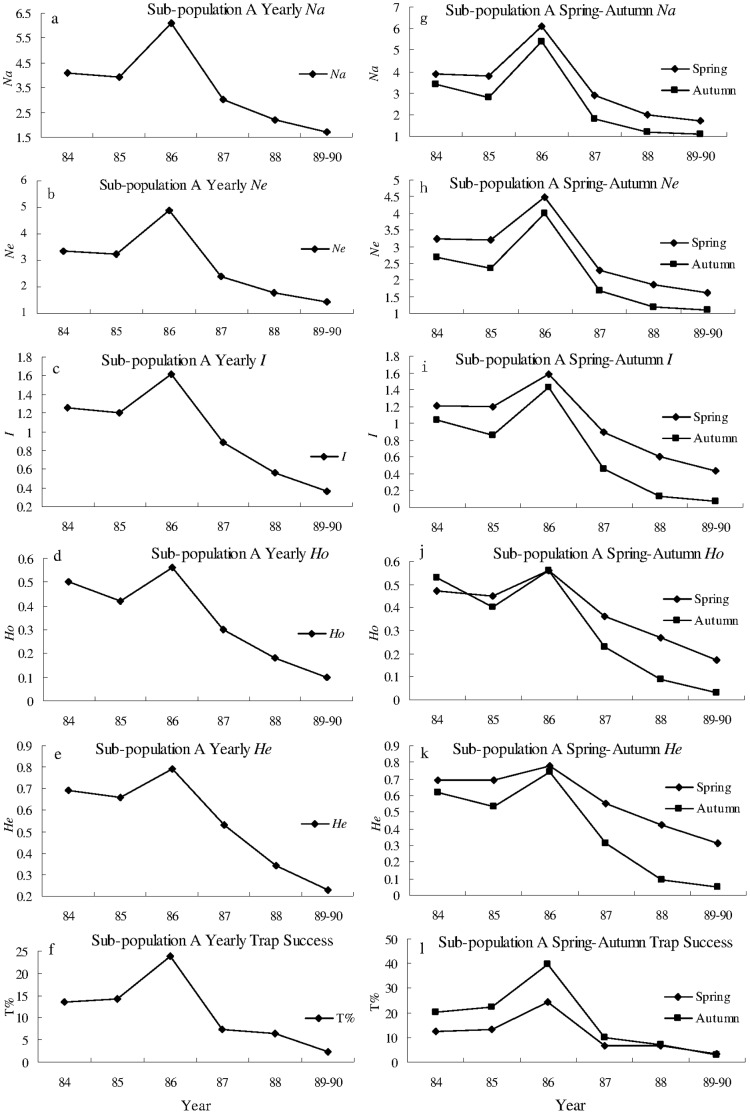
Genetic diversity and trap success of sub-population A from 1984 to 1990. a)–f) yearly, g)–l) spring and autumn genetic diversity (*Na*, *Ne*, *I*, *He*, and *Ho*) and trap success (T%) of sub-population A from 1984 to 1990. *Na*: the observed average number of alleles per locus. *Ne*: the effective average number of alleles per locus. *I*: Shannon’s Information index. *He*: unbiased estimates of expected heterozygosities. *Ho*: observed heterozygosities.

**Figure 3:**
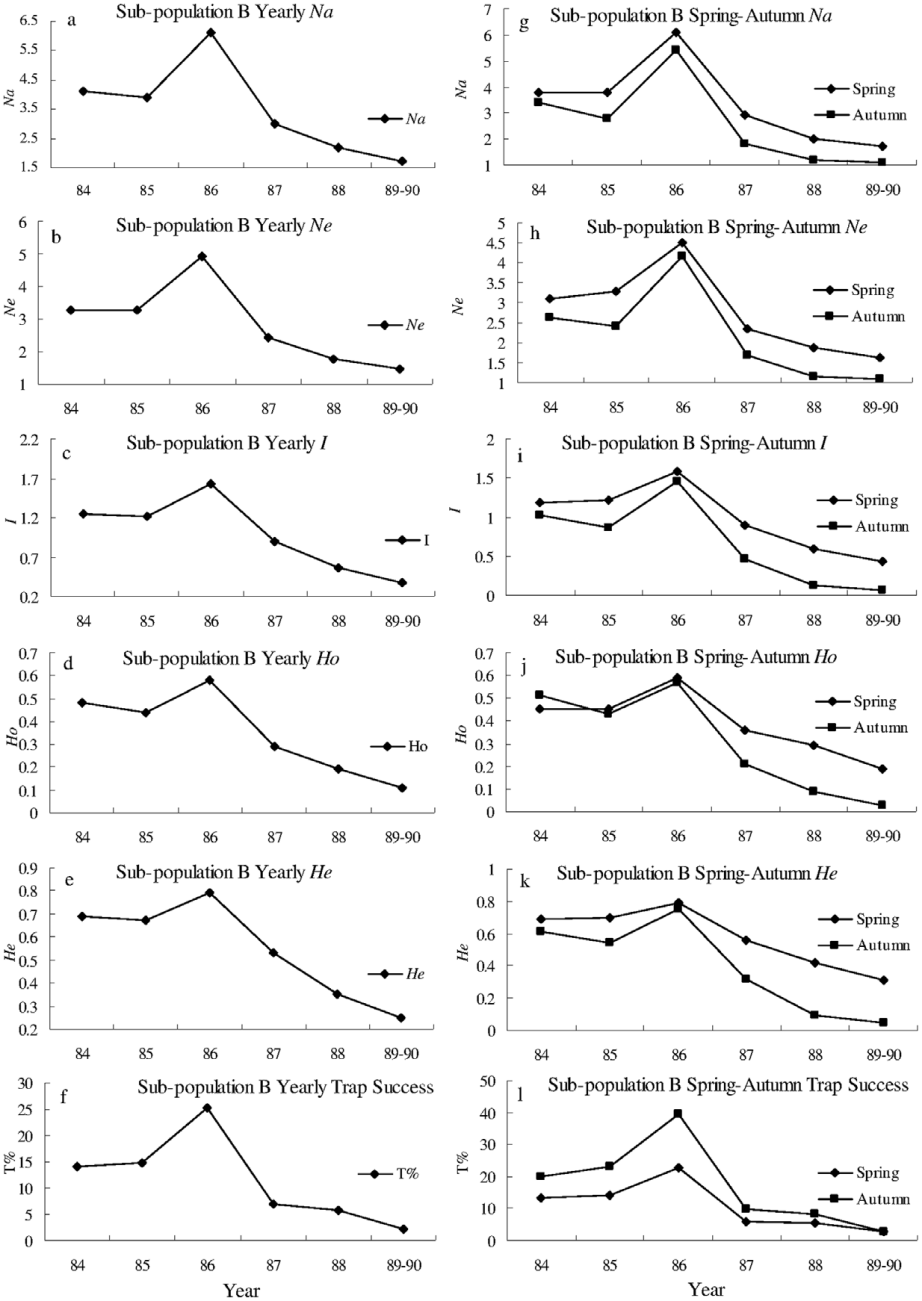
Genetic diversity and trap success of sub-population B from 1984 to 1990. a)–f) yearly, g)–l) spring and autumn genetic diversity (*Na*, *Ne*, *I*, *He,* and *Ho*) and trap success (T%) of sub-population B from 1984 to 1990. *Na*, *Ne*, *I*, *He*, and *Ho*: see figure 2.

**Figure 4:**
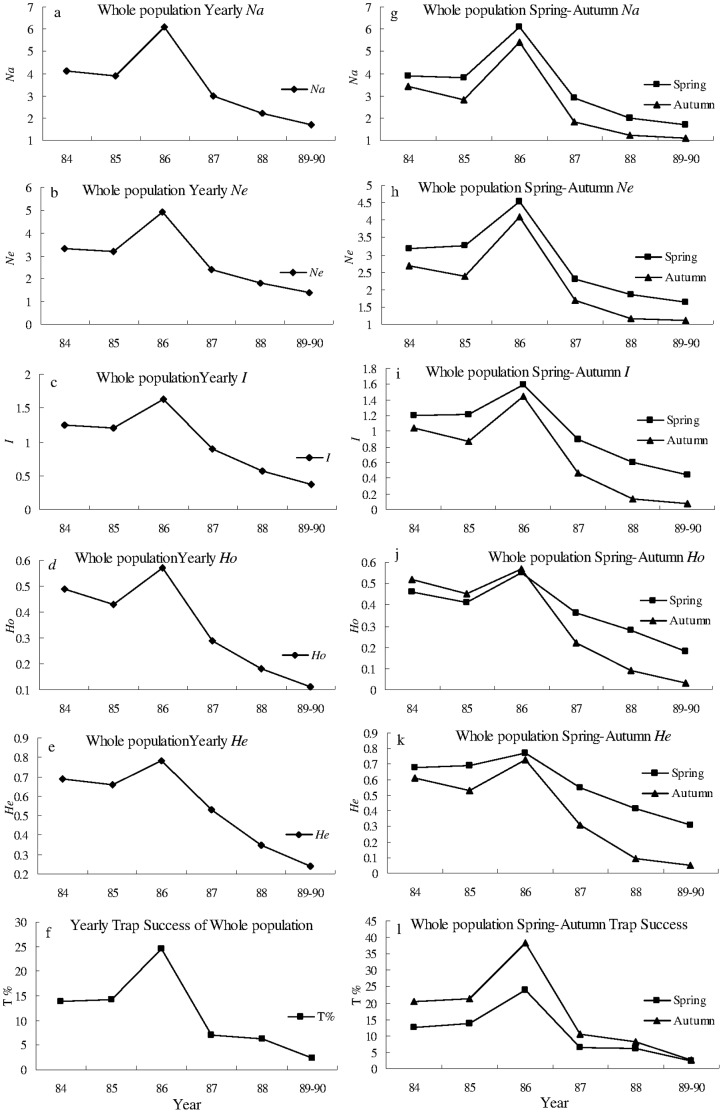
Genetic diversity and trap success of the whole population from 1984 to 1990. a)–f) yearly, g)–l) spring and autumn genetic diversity (*Na*, *Ne*, *I*, *He*, and *Ho*) and trap success (T%) of the whole population from 1984 to 1990. *Na*, *Ne*, *I*, *He*, and *Ho*: see figure 2.

There was a large irruption of the Greater long-tailed hamster in the Raoyang region during the 1980’s. The population reached peak in 1986, and then showed steady decline until 1990 (Figure 2 f; Figure 3 f; Figure 4 f). All seasonal or yearly genetic diversity indices showed similar trends to that of population density (trap success, %) during 1984–1990 for the whole population, and sub-population A and B. No locus was found to deviate significantly from the Hardy-Weinberg equilibrium within each of the three populations.

For sub-population A, B and the whole population, *Na*, *Ne*, *I*, *He*, and *Ho* were all significantly positively correlated with trap success in spring, autumn and yearly (Figure 2, 3, 4 and Table 1), except *He* was not significantly correlated with trap success in spring (*r = *0.902, *P = *0.701) for the whole population. For example, *I* was significantly (*P* = 0.005, 0.002 and 0.001) and positively (r = 0.910, 0.962 and 0.964) correlated with trap success in spring, autumn, and yearly, respectively (Figure 2 and Table 1). These results indicate that genetic diversity is density-dependent in a rapidly expanding population of the Greater long-tailed hamster.

**Table 1:** Pearson correlations between genetic diversity (*Na*, *Ne*, *I*, *He*, and *Ho*) and trap success (T%) of the Greater long-tailed hamster (sub-population A, B and the whole population) in spring, autumn and the whole year.

		Correlation coefficients		
		Sub-population A	Sub-population B	The whole population
Spring	*Na*	*r = *0.980	*r = *0.981	*r = *0.984
		*P = *0.005**	*P = *0.001**	*P = *0.001**
	*Ne*	*r = *0.981	*r = *0.992	*r = *0.976
		*P = *0.005 **	*P = *0.001**	*P = *0.001**
	*I*	*r_s_ = *0.942	*r = *0.963	*r = *0.957
		*P = *0.005**	*P = *0.002**	*P = *0.002**
	*Ho*	*r = *0.910	*r = *0.964	*r = *0.938
		*P = *0.012*	*P = *0.002**	*P = *0.005**
	*He*	*r = *0.884	*r = *0.917	*r = *0.902
		*P = *0.019*	*P = *0.010*	*P = *0.701
Autumn	*Na*	*r = *0.982	*r = *0.975	*r = *0.978
		*P = *0.004**	*P = *0.001**	*P = *0.001**
	*Ne*	*r = *0.983	*r = *0.982	*r* = 0.981
		*P = *0.004**	*P = *0.001**	*P = *0.001**
	*I*	*r = *0.962	*r = *0.957	*r = *0.965
		*P = *0.002**	*P = *0.002**	*P = *0.002**
	*Ho*	*r = *0.900	*r = *0.910	*r = *0.906
		*P = *0.015*	*P = *0.012*	*P = *0.013*
	*He*	*r = *0.928	*r = *0.923	*r = *0.928
		*P = *0.008**	*P = *0.009**	*P = *0.008**
Yearly	*Na*	*r = *0.990	*r = *0.990	*r = *0.982
		*P = *0.001**	*P = *0.001**	*P = *0.001**
	*Ne*	*r = *0.990	*r = *0.993	*r = *0.985
		*P = *0.001**	*P = *0.001**	*P = *0.001**
	*I*	*r = *0.964	*r = *0.965	*r = *0.952
		*P = *0.001**	*P = *0.002**	*P = *0.003**
	*Ho*	*r = *0.931	*r = *0.955	*r = *0.938
		*P = *0.007**	*P = *0.003**	*P = *0.006**
	*He*	*r = *0.937	*r = *0.921	*r = *0.906
		*P = *0.006**	*P = *0.009**	*P = *0.013*

** Correlation is significant at the 0.01 level (2-tailed).

* Correlation is significant at the 0.05 level (2-tailed).

The values of *Na*, *Ne*, *I*, *Ho* and *He* in spring tended to be higher than values in autumn for sub-population A and B, and the whole population, except in 1984, when *Ho* in spring was lower than one in autumn for sub-population A and B. A paired t-test indicated that *Na*, *Ne*, *I* and *He* were significantly larger in spring than in autumn (*P*<0.01); trap success (T%) was also significantly lower in spring than in autumn (*P*<0.05) for sub-population A, sub-population B and the whole population, except *Ho* in spring was not significantly different from that in autumn (Table S2). These results indicate that genetic diversity is higher and density is lower in spring than in autumn for a rapidly expanding population of the Greater long-tailed hamster.

Yearly *Na*, *Ne*, *I*, *Ho* and *He* were all significantly and positively (*P*<0.01) correlated with yearly trap success for sub-population A and B (Figure 2, Figure 3 and Table 1). Significant and positive correlation (*P*<0.01) was found between yearly *Na*, *Ne*, *I*, and *Ho* and yearly trap success for the whole population (Figure 4 and Table 1). Positive and significant correlation (*P*<0.05) existed between yearly *He* and yearly trap success for the whole population (Figure 4 and Table 1). These results indicate that the genetic diversity is positively correlated with population density in a rapidly expanding population of the Greater long-tailed hamster.

**Figure 5:**
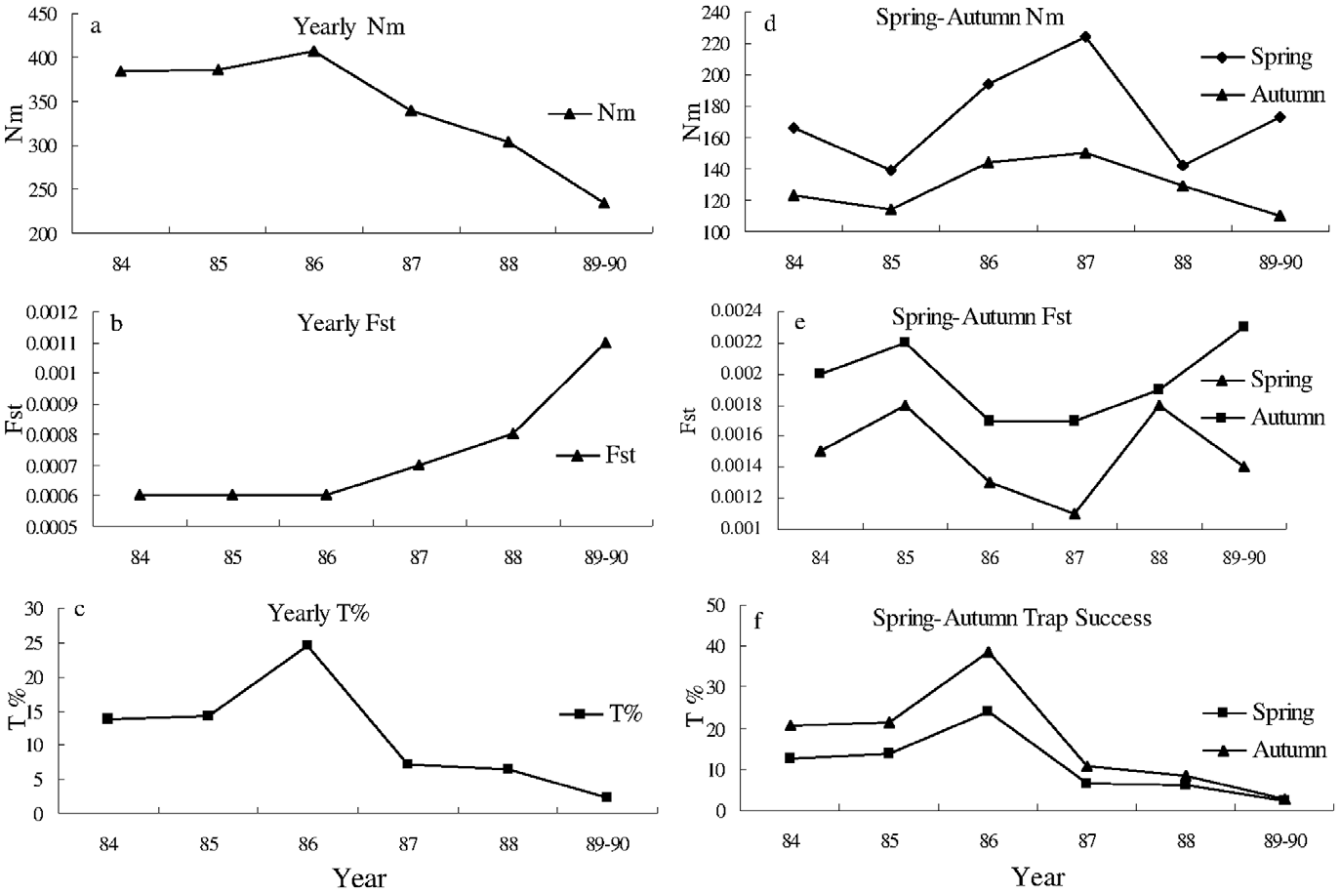
Gene flow (Nm) and genetic differentiation (Fst) between sub-population A and B and trap success (T%) of the whole population from 1984 to 1990. a) and b) yearly, d) and e) spring and autumn gene flow (Nm) and genetic differentiation (Fst) of the Greater long-tailed hamster between sub-population A and B, and c) yearly, f) spring and autumn trap success (T%) of the whole population from 1984 to 1990.

As shown in Figure 5 and Table S3, yearly gene flow (Nm) between sub-population A and B was positively and significantly associated with yearly trap success (*r* = 0.879, *P* = 0.021). Negative association (*r* = −0.764) between genetic differentiation (Fst) and yearly trap success with an approximate significance level (*P = *0.077) was found between sub-population A and B. Such correlations for spring and autumn populations were non-significant (Table S3), which indicates that the season also plays important role for gene flow. Thus, the results suggest that both population density and season influence gene flow and genetic diversity in a rapidly expanding population of the Greater long-tailed hamster.

The years 1984, 1985 and 1986 were defined as high density years (average yearly trap success = 19.2%), while the years 1987, 1988 and 1989–1990 were low density years (average yearly trap success = 4.8%). In high density years, we found that gene flow between sub-population A and B (Nm = 522.7) was higher than in low density years (Nm = 312.4), and genetic differentiation (Fst = 0.0005) was lower than in low density years (Fst = 0.0008). These results indicate that dispersal is density-dependent for a rapidly-expanding population of the Greater long-tailed hamster.

## Discussion

Our results show that, on a multi-annual scale, genetic diversity (spring, autumn, and yearly) of the Greater long-tailed hamsters is higher in high-density years than in low-density years. This observation is consistent with previous studies indicating that genetic diversity of this species is positively density-dependent [13], [14], [26]. Our previous studies were all based on data collected in years of low- or medium-sized population density. Our current results suggest that in a rapidly expanding population of this species, the positive density-dependent rule of population genetic diversity still holds.

Dong *et al* (2010) proposed a hypothesis to explain the observed positive association between genetic diversity and population density in this hamster species [13]. This species is solitary and very aggressive, and in high density, mate competition, reduced home range and shortage of resources would increase individual dispersal rate, which in turn increases gene flow and the genetic diversity of the population [14], [27]–[29]. For example, in this study, yearly gene flow between sub-population A and B of the Greater long-tailed hamster is positively correlated with yearly trap success during 1984–1990 (Figure 5 and Table S3). When population density is low, dispersal-driven gene flow becomes low. Variation of genetic diversity in different density-phases may affect population dynamics. In high-density phases, relatedness between individuals would be low due to wider dispersal of individuals, which may increase social conflicts, stress and mortality [14]. In low-density phases, inbreeding and genetic drift may occur, causing low genetic diversity, which may not favor population recovery. Our data show that the population of Greater long-tailed hamsters often experienced several low-density years after irruptions. For example, the population density was very low since 1990 (1.8%) until 1996 when it reached 1.5%, then it had increased somewhat from 1997 (2.9%). The low population density has been revealed to potentially lead to genetic bottlenecks in *T. triton* [13]. Therefore, we inferred that low or extreme low genetic diversity in Greater long-tailed hamsters may attribute to sustain low density of the population.

This positive density-dependent dispersal pattern of *T. triton* is very different from that of some voles and several other taxa. Emerging empirical evidence indicates that negative density-dependent dispersal is prevalent in voles [30]–[32], kangaroo rats [33] and in several other taxa [34]–[37]. The different density-dependent dispersal patterns between voles and hamsters lie in their life history and how they interact with the environment [38]. For example, voles like social life, while the Greater long-tailed hamster is a typical solitary species. In voles, both scarcity of mates and inbreeding avoidance may result in greater dispersal at low population density. Moreover, reduced dispersal at high densities may result from suppressed sexual maturation in voles [39]. Whereas in the Greater long-tailed hamsters, mate competition, reduced home range and shortage of resources at high population density may result in increased dispersal, while low dispersal in low population density may increase the probability of mating success, and eventually lead to increasing population size. Thus, we inferred that living habits may be a main factor affecting the correlations between the population density and dispersal.

It is interesting that we found a reversed relationship between genetic diversity and population density within the annual scale; population density tended to be lower in spring than in autumn, but genetic diversity tended to be higher in spring than in autumn. For example, the Greater long-tailed hamsters had significant lower density for sub-population A (*P = *0.032), sub-population B (*P* = 0.021), and the whole population (*P* = 0.016) in spring than in autumn (Table S2); while the Greater long-tailed hamsters had significant higher gene flow (*P* = 0.002) between sub-population A and B in spring than in autumn (Table S4). Higher gene flow increased the relatedness of the two sub-populations, so sub-population A and B has significant lower Fst and higher genetic identity in spring than in autumn (Table S4 and Figure 5). We previously proposed a season-dependent dispersal hypothesis to explain the observed phenomenon [26]. The hamster species usually breeds from April to August, and its population often reaches its peak in autumn. In spring, dispersal rate would be high as individuals seek mates, then relatedness between individuals would be low. This inbreeding avoidance would benefit the population. In autumn, by the end of breeding season, young newborn hamsters appear nearby parent burrows, which may increase the relatedness and decrease genetic diversity of autumn populations. Young hamsters need to build their burrows and store enough food for over-wintering. Dispersal rate is low until spring when breeding begins. Our results support the season-dependent dispersal hypothesis proposed by Wang *et al* [26].

It is notable that in the expansion period, the observed rule is not dependent on spatial scale. High dispersal between sub-population A and B existed for a rapidly-expanding population of the Greater long-tailed hamsters. The activity range of these hamsters is 100–300 meters [40]. The Jingjiu railway and S282 Road had less effect on dispersal, which is discrepancy with the previous studies [15]–[17]. This discrepancy may be caused by high dispersal which is contributed by high population density, large dispersal distance, and frequent agricultural activities. On the other hand, the high population density and large activity range play the crucial roles in high dispersal which is intensified by frequent agricultural activities such as irrigation, ploughing, etc. These agricultural activities often destroy burrow systems, forcing hamsters to migrate frequently, and resulting in high dispersal rates. Thus, further studies are expected to further explore the mechanisms that decrease the effects of the roads as geographical barriers on population dispersal.

## Supporting Information

Table S1 Primer sequences of ten microsatellites used for studying genetic diversity of the Greater long-tailed hamsters.
(DOC)

Table S2 T- test of genetic diversity (*Na*, *Ne*, *I*, *He*, and *Ho*) and trap success (T%) between spring and autumn populations for sub-population A, B and the whole population.
(DOC)

Table S3 Pearson correlations between genetic parameters and trap success (T%) of the whole population in spring, autumn and whole year. Genetic parameters refer to Nm and Fst respectively, between sub-population A and B.
(DOC)

Table S4 T- test for the genetic parameters between spring and autumn populations. Genetic parameters refer to Nm and Fst, respectively, between sub-population A and B.
(DOC)
