# Supplementary material for: Variation of Genetic Diversity in a Rapidly Expanding Population of the Greater Long-Tailed Hamster (Tscherskia triton) as Revealed by Microsatellites
Source: PLoS One. 2013 Jan 17;8(1):e54171. doi: 10.1371/journal.pone.0054171 (PMC3547878; doi:10.1371/journal.pone.0054171)
Supplement: Table S1 — Primer sequences of ten microsatellites used for studying genetic diversity of the Greater long-tailed hamsters. (DOC) [file pone.0054171.s001.doc]

Table S1

| Locus | GenBank Accession no. | Primer sequences (5′−3′; F, forward; R, reverse) | Repeat motif | Size range  (bp) | *T*a |
| --- | --- | --- | --- | --- | --- |
| GYA66 | AY780301 | F: CCCAGGAATGTTTATC | (CT)_25_(GT)_21_ | 435-474 | 53 |
|  |  | R: AAGCCACCTTACTGACCC |  |  |  |
| GYA136 | AY780304 | F: CAGTCAGCCTTGTTCCAG | (CT)_4_(CA)_4_(TG)(CA)(TA)_2_(CA)C(CA)_4_(TA)(CA)T(TA)(CA)(CAA)_4_ | 148-185 | 54 |
|  |  | R: CAAATGCCCTCTTAGTGT |  |  |  |
| GYA183 | AY780297 | F: GAACTGATGCCCTTGTGG | (CA)_4_ | 339-368 | 51 |
|  |  | R: CATTCCCTTATTGTCTGG |  |  |  |
| GYA189 | AY780309 | F: AAACATAAATGGGAGACA | (AC)_16_(AG)_10_(A)_2_  (AG)_10_ | 253-275 | 55 |
|  |  | R: CTAAACCTGAACTGAGC |  |  |  |
| GYB13 | AY864068 | F: ATGAAGGTAGAAAGAGGGAA | （CA）_20_ | 114-146 | 47 |
|  |  | R: TTATGAGTGGGGTGCTGA |  |  |  |
| GYB47 | AY864074 | F: ATCCCTCTTCTCTCTTCTGG | （CA）_18_（GA）_20_ | 291-338 | 54 |
|  |  | R: AAAGCACTACTACCTCTGA |  |  |  |
| GYA185 | AY780308 | F: AAACAGGAACTATGGAGGCA | （AC）_14_ | 329-354 | 55 |
|  |  | R: TGGTATAATTTATTTGGTG |  |  |  |
| GY103 | AY780298 | F: CTGGTCCTCTGAAAAG | （GT）_16_ | 166-182 | 50 |
|  |  | R: AACCTACTGCCTCTAT |  |  |  |
| GYB28 | AY864071 | F: CCTCTGTCATCCCCAAGT | （CCTT）_11_ | 331-383 | 51 |
|  |  | R: AGAAACCCTGTCTCAAAA |  |  |  |
| GYA181 | AY780305 | F: GGGCTGACTTACAGTTTTAG | （GT）_15_（AT）（GT）_12_ | 169-185 | 51 |
|  |  | R: CAAGGTGGGCTTTGAGGT |  |  |  |

*Ta*: annealing temperature(ºC)
