# Supplementary material for: Variation of Genetic Diversity in a Rapidly Expanding Population of the Greater Long-Tailed Hamster (Tscherskia triton) as Revealed by Microsatellites
Source: PLoS One. 2013 Jan 17;8(1):e54171. doi: 10.1371/journal.pone.0054171 (PMC3547878; doi:10.1371/journal.pone.0054171)
Supplement: Table S2 — T- test of genetic diversity (Na, Ne, I, He, and Ho) and trap success (T%) between spring and autumn populations for sub-population A, B and the whole population. (DOC) [file pone.0054171.s002.doc]

**Table S2**

|  | sub-population A | sub-population B | The whole population |
| --- | --- | --- | --- |
| *Na* | 0.002** | 0.004** | 0.001** |
| *Ne* | 0.001** | 0.002** | 0.001** |
| *I* | 0.009** | 0.001** | 0.001** |
| *Ho* | 0.051 | 0.054 | 0.100 |
| *He* | 0.005** | 0.005** | 0.005** |
| T% | 0.032* | 0.021* | 0.016* |

** Differentiation is significant at the 0.01 level (2-tailed)

* Differentiation is significant at the 0.05 level (2-tailed)
