# Supplementary material for: Variation of Genetic Diversity in a Rapidly Expanding Population of the Greater Long-Tailed Hamster (Tscherskia triton) as Revealed by Microsatellites
Source: PLoS One. 2013 Jan 17;8(1):e54171. doi: 10.1371/journal.pone.0054171 (PMC3547878; doi:10.1371/journal.pone.0054171)
Supplement: Table S3 — Pearson correlations between genetic parameters and trap success (T%) of the whole population in spring, autumn and whole year. Genetic parameters refer to Nm and Fst respectively, between sub-population A and B. (DOC) [file pone.0054171.s003.doc]

**Table S3**

|  | Nm | Fst |
| --- | --- | --- |
| Spring | *r* = 0.071 | *r* = -0.051 |
|  | *P* = 0.894 | *P* = 0.924 |
| Autumn | *r* =0.348 | *r* =-0.441 |
|  | *P* =0.499 | *P* =0.382 |
| Yearly | *r_s_* = 0.879 | *r* = -0.764 |
|  | *P* = 0.021* | *P* = 0.077 |

** Correlation is significant at the 0.01 level (2-tailed)

* Correlation is significant at the 0.05 level (2-tailed)
