# Supplementary material for: Variation of Genetic Diversity in a Rapidly Expanding Population of the Greater Long-Tailed Hamster (Tscherskia triton) as Revealed by Microsatellites
Source: PLoS One. 2013 Jan 17;8(1):e54171. doi: 10.1371/journal.pone.0054171 (PMC3547878; doi:10.1371/journal.pone.0054171)
Supplement: Table S4 — T- test for the genetic parameters between spring and autumn populations. Genetic parameters refer to Nm and Fst, respectively, between sub-population A and B. (DOC) [file pone.0054171.s004.doc]

**Table S4**

|  | Nm | Fst |
| --- | --- | --- |
| *P* value | 0.002** | 0.003** |

** Differentiation is significant at the 0.01 level (2-tailed)

* Differentiation is significant at the 0.05 level (2-tailed)
